# Crystal structure, Hirshfeld surface analysis and DFT study of (2*Z*)-2-(4-fluoro­benzyl­idene)-4-(prop-2-yn-1-yl)-3,4-di­hydro-2*H*-1,4-benzo­thia­zin-3-one

**DOI:** 10.1107/S2056989019002354

**Published:** 2019-02-22

**Authors:** Brahim Hni, Nada Kheira Sebbar, Tuncer Hökelek, Younes Ouzidan, Ahmed Moussaif, Joel T. Mague, El Mokhtar Essassi

**Affiliations:** a Laboratoire de Chimie Organique Hétérocyclique URAC 21, Pôle de Compétence Pharmacochimie, Av. Ibn Battouta, BP 1014, Faculté des Sciences, Université Mohammed V, Rabat, Morocco; b Laboratoire de Chimie Bioorganique Appliquée, Faculté des Sciences, Université Ibn Zohr, Agadir, Morocco; c Department of Physics, Hacettepe University, 06800 Beytepe, Ankara, Turkey; d Laboratoire de Chimie Organique Appliquée, Université Sidi Mohamed Ben Abdallah, Faculté des Sciences et Techniques, Route d’immouzzer, BP 2202, Fez, Morocco; e National Center of Energy Sciences and Nuclear Techniques, Rabat, Morocco; f Department of Chemistry, Tulane University, New Orleans, LA 70118, USA; g Moroccan Foundation for Advanced Science, Innovation and Research (MASCIR), Rabat, Morocco

**Keywords:** crystal structure, di­hydro­benzo­thia­zine, hydrogen bond, DFT, Hirshfeld surface

## Abstract

The title compound, C_18_H_12_FNOS, is built up from a 4-fluoro­benzyl­idene moiety and a di­hydro­benzo­thia­zine unit with a propynyl substituent, with the heterocyclic portion of the di­hydro­benzo­thia­zine unit adopting a shallow boat conformation with the propynyl substituent nearly perpendicular to it. The two benzene rings are oriented at a dihedral angle of 43.02 (6)°. In the crystal, C—H_Flurphen_⋯F_Flurphen_ (Flurphen = fluoro­phen­yl) hydrogen bonds link the mol­ecules into inversion dimers, enclosing *R*_2_^2^(8) ring motifs, with the dimers forming oblique stacks along the *a*-axis direction. Hirshfeld surface analysis of the crystal structure indicates that the most important contributions to the crystal packing are from H⋯H (33.9%), H⋯C/C⋯H (26.7%), H⋯F/F⋯H (10.9%) and C⋯C (10.6%) inter­actions. Hydrogen bonding and van der Waals inter­actions are the dominant inter­actions in the crystal packing. Density functional theory (DFT) optimized structures at the B3LYP/6–311 G(d,p) level are compared with the experimentally determined mol­ecular structure in the solid state. The HOMO–LUMO behaviour was elucidated to determine the energy gap.

## Abstract 2

In the title compound, the heterocyclic portion of the di­hydro­benzo­thia­zine unit adopts a shallow boat conformation. The propynyl substituent is nearly perpendicular to the plane formed by the rails of the boat. In the crystal, inversion dimers are formed by weak C—H⋯F hydrogen bonds with the dimers forming oblique stacks along the *a*-axis direction.

## Chemical context   

1,4-Benzo­thia­zine derivatives represent one of the most important classes of organic mol­ecules and have been studied extensively for their biological activities (Ellouz *et al.*, 2017*a* ▸; Sebbar *et al.*, 2016*a* ▸) and therapeutic applications such as analgesic (Wammack *et al.*, 2002 ▸), anti-viral (Malagu *et al.*, 1998 ▸; Rathore & Kumar, 2006 ▸) and anti-oxidant activities (Zia-ur-Rehman *et al.*, 2009 ▸). Slight changes in the substitution pattern in the benzo­thia­zine nucleus can cause a distinguishable difference in their biological properties (Niewiadomy *et al.*, 2011 ▸; Armenise *et al.*, 2012 ▸). Recent research has been focused on existing mol­ecules and their modifications in order to reduce their side effects and to explore their other pharmacological and biological effects (Ellouz *et al.*, 2017*b* ▸; Sebbar *et al.*, 2016*b* ▸; Gautam *et al.*, 2012 ▸). As a continuation of our research into the development of N-substituted 1,4-benzo­thia­zine derivatives and the evaluation of their potential pharmacological activities, we have studied the condensation reaction of propargyl bromide with (*Z*)-2-(4-fluoro­benzyl­idene)-2*H*-1,4-benzo­thia­zin-3(4*H*)-one under phase-transfer catalysis conditions using tetra-*n*-butyl­ammonium bromide (TBAB) as catalyst and potassium carbonate as base, leading to the title compound namely (2*Z*)-2-(4-fluoro­benzyl­idene)-4-(prop-2-yn-1-yl)-3,4-di­hydro-2*H*-1,4-benzo­thia­zin-3-one in good yield (Sebbar *et al.*, 2017*a* ▸, Ellouz *et al.*, 2018 ▸), and we report herein its synthesis, the mol­ecular and crystal structures, along with the Hirshfeld surface analysis and density functional theory (DFT) computational calculations carried out at the B3LYP/6–311 G(d,p) level.
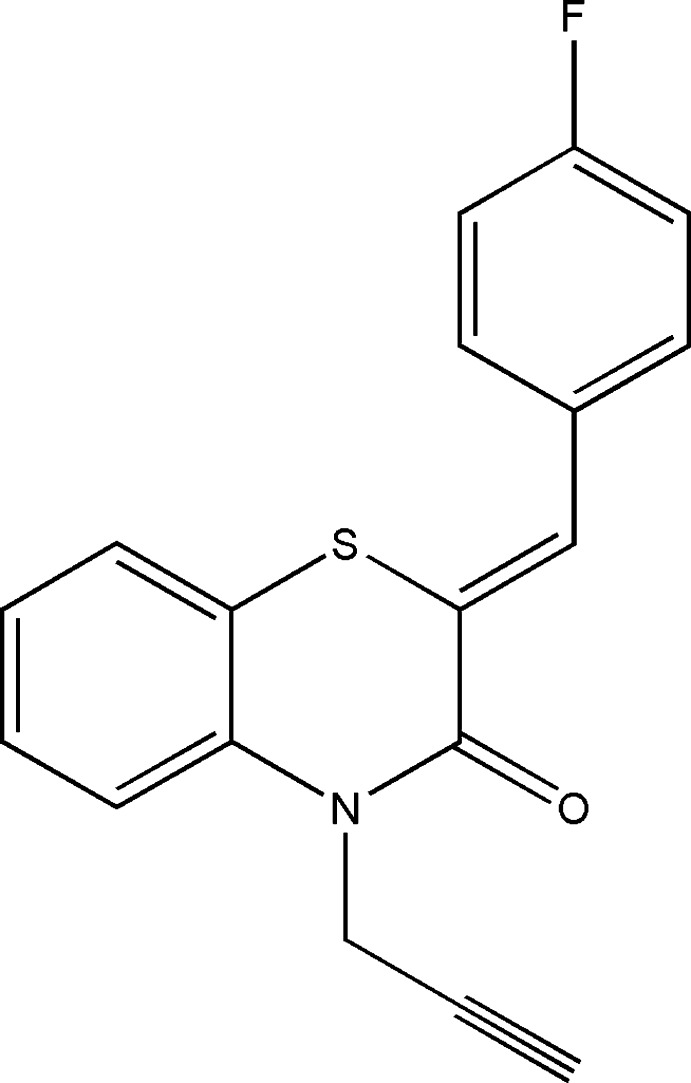


## Structural commentary   

**Figure 1:**
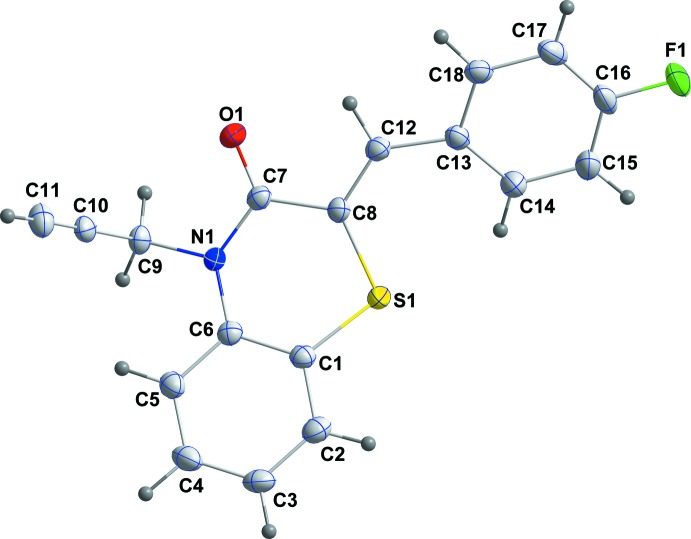
The mol­ecular structure of the title compound with the atom-numbering scheme. Displacement ellipsoids are drawn at the 50% probability level.

The title compound, (I), is built up from a 4-fluoro­phenyl­methyl­idene moiety and a di­hydro­benzo­thia­zine unit with a propynyl substituent (Fig. 1 ▸). The benzene (*A*; C1–C6), ring is oriented at a dihedral angle of 43.02 (6)° with respect to the 4-fluorophenyl ring (*C*; C13–C18). The propynyl substituent is nearly perpendicular to the plane defined by C1, C6, C7 and C8, as shown by the C6—N1—C9—C10 torsion angle of 81.3 (2)°. A puckering analysis of the heterocyclic ring (*B*; S1/N1/C1/C6–C8) of the di­hydro­benzo­thia­zine unit shows that it adopts a shallow boat conformation with puckering parameters *Q*_T_ = 0.3759 (14) Å, *q*_2_ = 0.3639 (15) Å, *q*_3_ = −0.0938 (17) Å, φ = 173.6 (3)° and θ = 104.5 (3)°. In the heterocyclic ring *B*, the C1—S1—C8 [101.73 (8)°], S1—C8—C7 [119.93 (12)°], C8—C7—N1 [119.23 (14)°], C7—N1—C6 [125.59 (14)°] and C6—C1—S1 [122.07 (13)°] bond angles are enlarged, while the N1—C6—C1 [120.91 (15)°] bond angle is narrowed when compared with the corresponding values in the closely related compounds 4-methyl-3,4-di­hydro-2*H*-1,4-benzo­thia­zin-3-one, (II) (Ellouz *et al.*, 2017*b* ▸), 4-[(3-phenyl-4,5-di­hydro­isoxazol-5-yl) meth­yl]-2*H-*benzo[*b*][1,4]thia­zin-3(4*H*)-one, (III) (Sebbar *et al.*, 2016*a* ▸) and (*Z*)-2-(2-chloro­benzyl­idene)-4-(prop-2-yn­yl)-2*H*-1,4-benzo­thia­zin-3(4*H*)-one, (IV), (Sebbar *et al.*, 2017*a* ▸).

## Supra­molecular features   

**Figure 2:**
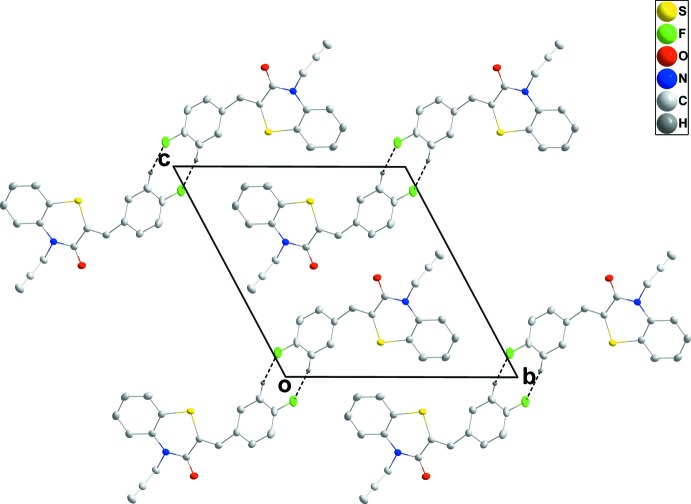
A partial packing diagram viewed along the *a*-axis direction. The inter­molecular C—H_Flurphen_⋯F_Flurphen_ (Flurphen = fluoro­phen­yl) hydrogen bonds are shown as dashed lines.

In the crystal, C—H_Flurphen_⋯F_Flurphen_ (Flurphen = fluoro­phen­yl) hydrogen bonds (Table 1 ▸) link the mol­ecules into inversion dimers enclosing 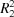
(8) ring motifs, with the dimers forming oblique stacks along the *a*-axis direction (Figs. 2 ▸ and 3 ▸).

## Hirshfeld surface analysis   

**Figure 4:**
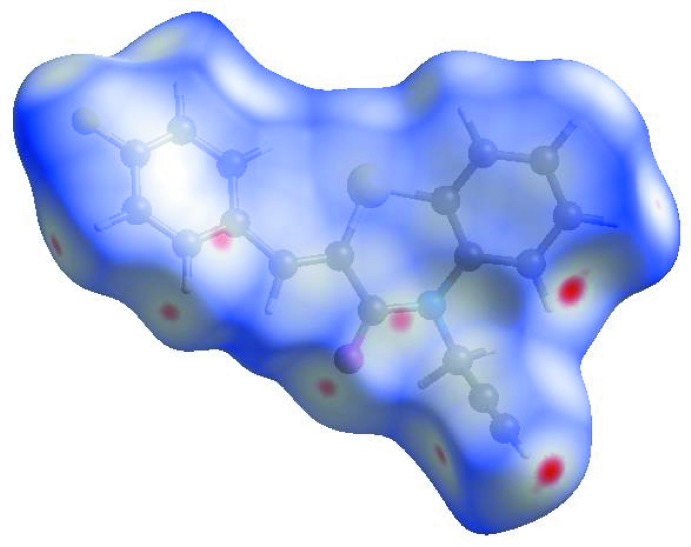
View of the three-dimensional Hirshfeld surface of the title compound plotted over *d*_norm_ in the range −0.0943 to 1.2826 a.u.

**Figure 5:**
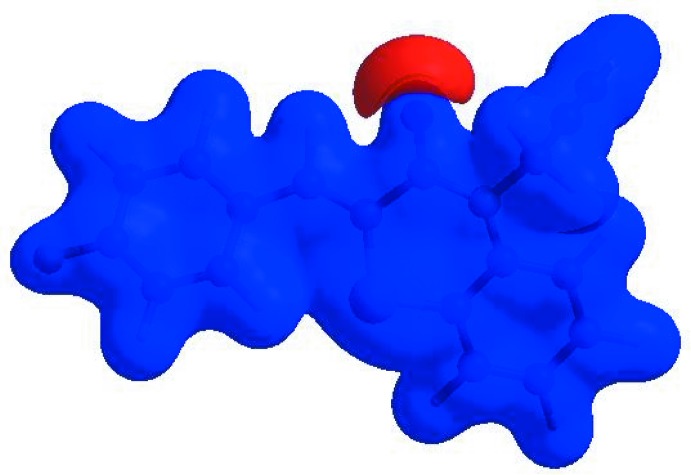
View of the three-dimensional Hirshfeld surface of the title compound plotted over electrostatic potential energy in the range −0.0500 to 0.0500 a.u. using the STO-3 G basis set at the Hartree–Fock level of theory. Hydrogen-bond donors and acceptors are shown as blue and red regions around the atoms corresponding to positive and negative potentials, respectively.

**Figure 6:**
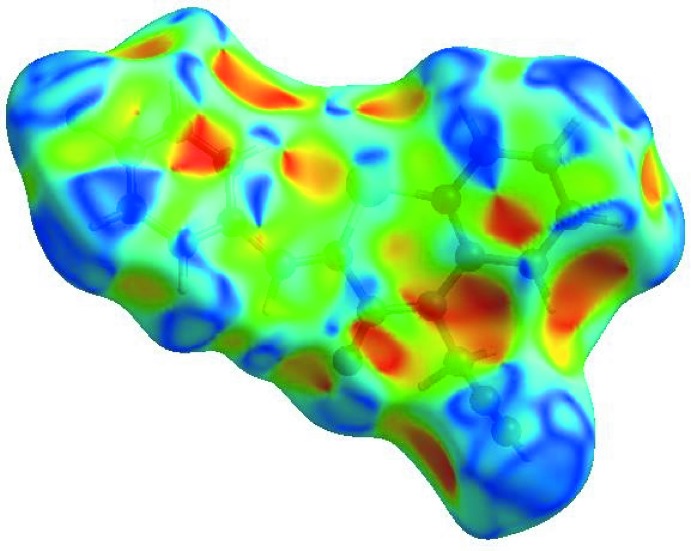
Hirshfeld surface of the title compound plotted over shape-index.

In order to visualize the inter­molecular inter­actions in the crystal of the title compound, a Hirshfeld surface (HS) analysis (Hirshfeld, 1977 ▸; Spackman & Jayatilaka, 2009 ▸) was carried out by using *CrystalExplorer17.5* (Turner *et al.*, 2017 ▸). In the HS plotted over *d*_norm_ (Fig. 4 ▸), the white surface indicates contacts with distances equal to the sum of van der Waals radii, and the red and blue colours indicate distances shorter (in close contact) or longer (distinct contact) than the van der Waals radii, respectively (Venkatesan *et al.*, 2016 ▸). The bright-red spots indicate their roles as the respective donors and/or acceptors; they also appear as blue and red regions corres­ponding to positive and negative potentials on the HS mapped over electrostatic potential (Spackman *et al.*, 2008 ▸; Jayatilaka *et al.*, 2005 ▸) as shown in Fig. 5 ▸. The blue regions indicate the positive electrostatic potential (hydrogen-bond donors), while the red regions indicate the negative electrostatic potential (hydrogen-bond acceptors). The shape-index of the HS is a tool to visualize the π–π stacking by the presence of adjacent red and blue triangles; if there are no adjacent red and/or blue triangles, then there are no π– π inter­actions. Fig. 6 ▸ clearly suggest that there are no π– π inter­actions in (I).

**Figure 7:**
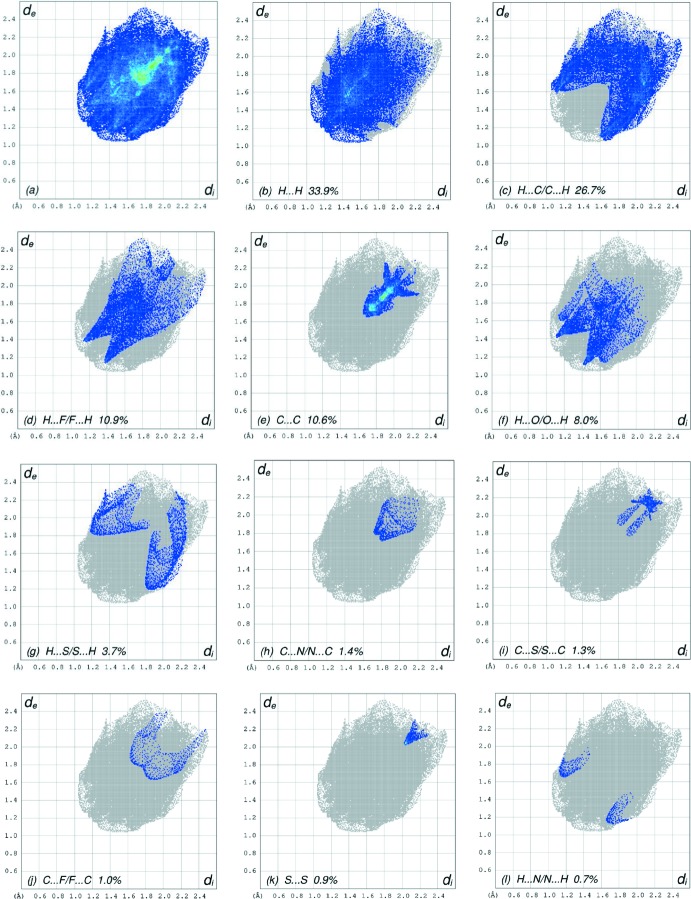
The full two-dimensional fingerprint plots for the title compound, showing (*a*) all inter­actions, and delineated into (*b*) H⋯H, (*c*) H⋯C/C⋯H, (*d*) H⋯F/F⋯H, (*e*) C⋯C, (*f*) H⋯O/O⋯H, (*g*) H⋯S/S⋯H, (*h*) C⋯N/N⋯C, (*i*) C⋯S/S⋯C, (*j*) C⋯F/F⋯C, (*k*) S⋯S and (*l*) H⋯N/N⋯H inter­actions. The *d*_i_ and d_e_ values are the closest inter­nal and external distances (in Å) from given points on the Hirshfeld surface.

**Figure 8:**
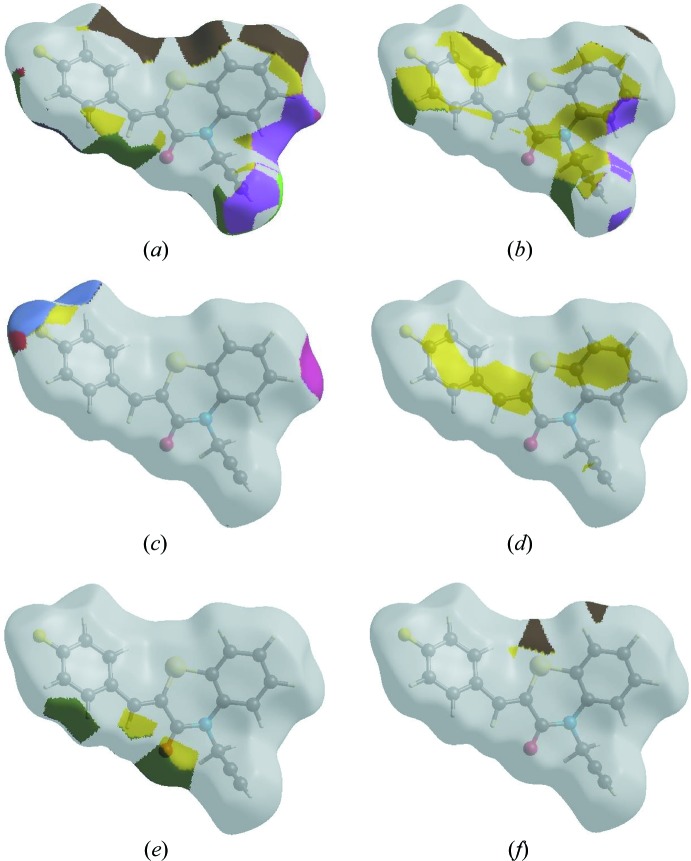
The Hirshfeld surface representations with the function *d*_norm_ plotted onto the surface for (*a*) H⋯H, (*b*) H⋯C/C⋯H, (*c*) H⋯F/F⋯H, (*d*) C⋯C, (*e*) H⋯O/O⋯H and (*f*) H⋯S/S⋯H inter­actions.

The overall two-dimensional fingerprint plot, Fig. 7 ▸ *a*, and those delineated into H⋯H, H⋯C/C⋯H, H⋯F/F⋯H, C⋯C, H⋯O/O⋯H, H⋯S/S⋯H, C⋯N/N⋯C, C⋯S/S⋯C, C⋯F/F⋯C, S⋯S and H⋯N/N⋯H contacts (McKinnon *et al.*, 2007 ▸) are illustrated in Fig. 7 ▸ *b*–*l*, respectively, together with their relative contributions to the Hirshfeld surface. The most important inter­action is H⋯H contributing 33.9% to the overall crystal packing, which is reflected in Fig. 7 ▸ *b* as widely scattered points of high density due to the large hydrogen content of the mol­ecule. In the absence of C—H⋯π inter­actions, the pair of scattered wings in the fingerprint plot delineated into H⋯C/C⋯H contacts (26.7% contribution to the HS) have a nearly symmetrical distribution of points, Fig. 7 ▸ *c*, with the thick edges at *d*_e_ + *d*_i_ ∼2.70 Å. The pair of characteristic wings in the fingerprint plot delineated into H⋯F/F⋯H contacts (Fig. 7 ▸ *d*, the 10.9% contribution to the HS) arises from the C—H⋯F hydrogen bonds (Table 1 ▸) as well as from the H⋯F/F⋯H contacts (Table 2 ▸) and is shown as a pair of spikes with the tips at *d*_e_ + *d*_i_ = 2.52 Å. The C⋯C contacts (Fig. 7 ▸ *e*, 10.6% contribution to the HS) have an arrow-shaped distribution of points with the tip at *d*_e_ = *d*_i_ ∼1.68 Å. The pair of characteristic wings in the fingerprint plot delineated into H⋯O/O⋯H contacts (Fig. 7 ▸ *f*, 8.0% contribution to the HS) have a pair of spikes with the tips at *d*_e_ + *d*_i_ = 2.54 Å. Finally, the H⋯S/S⋯H contacts (Table 2 ▸; Fig. 7 ▸ *g*, 3.7% contribution) are viewed as A pair of wide spikes with the tips at *d*_e_ + *d*_i_ = 3.02 Å. The Hirshfeld surface representations with the function *d*_norm_ plotted onto the surface are shown for the H⋯H, H⋯C/C⋯H, H⋯F/F⋯H, C⋯C, H⋯O/O⋯H and H⋯S/S⋯H inter­actions in Fig. 8 ▸ *a*–*f*, respectively.

The Hirshfeld surface analysis confirms the importance of H-atom contacts in establishing the packing. The large number of H⋯H, H⋯C/C⋯H and H⋯O/O⋯H inter­actions suggest that van der Waals inter­actions and hydrogen bonding play the major roles in the crystal packing (Hathwar *et al.*, 2015 ▸).

## DFT calculations   

**Figure 9:**
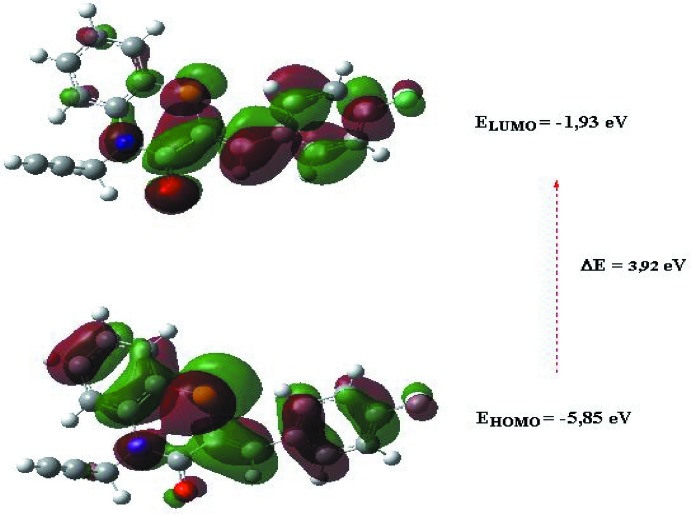
The energy band gap of the title compound.

The optimized structure of the title compound, (I), in the gas phase was generated theoretically *via* density functional theory (DFT) using standard B3LYP functional and 6–311G(d,p) basis-set calculations (Becke, 1993 ▸) as implemented in *GAUSSIAN 09* (Frisch *et al.*, 2009 ▸). The theoretical and experimental results were in good agreement. The highest-occupied mol­ecular orbital (HOMO), acting as an electron donor, and the lowest-unoccupied mol­ecular orbital (LUMO), acting as an electron acceptor, are very important parameters for quantum chemistry. When the energy gap is small, the mol­ecule is highly polarizable and has high chemical reactivity. The electron transition from the HOMO to the LUMO energy level is shown in Fig. 9 ▸. The HOMO and LUMO are localized in the plane extending from the whole (*Z*)-2-(4-fluoro­benzyl­idene)-4-(prop-2-yn­yl)-2*H*-1,4-benzo­thia­zin-3(4*H*)-one ring. The energy band gap [Δ*E* = *E*_LUMO_ - *E*_HOMO_] of the mol­ecule was about 3.92 eV, and the frontier mol­ecular orbital energies, E_HOMO_ and E_LUMO_ were −5.85 and −1.93 eV, respectively.

## Database survey   

Using the search fragment **II** (*R*_1_ = Ph, *R*_2_ = C) in the Cambridge Crystallographic Database (Groom *et al.*, 2016 ▸; updated to Nov. 2018), 14 hits were registered with *R*_1_ = Ph and *R*_2_ = CH_2_COOH (Sebbar *et al.*, 2016*c* ▸), **IIa** (Sebbar *et al.*, 2016*b* ▸), *n*-octa­decyl (Sebbar *et al.*, 2017*b* ▸), **IIb** (Ellouz *et al.*, 2015 ▸), *n*-Bu (Sebbar, El Fal *et al.*, 2014 ▸), **IIc** (Sebbar *et al.*, 2016*d* ▸), **IId** (Sebbar *et al.*, 2015 ▸), CH_2_C≡CH **IIe** (Sebbar, Zerzouf *et al.*, 2014 ▸). In addition there are examples with *R*_1_ = 4-ClC_6_H_4_ and *R*_2_ = CH_2_Ph2 (Ellouz *et al.*, 2016 ▸) **IIf** and *R*_1_ = 2-ClC_6_H_4_, *R*_2_ = CH_2_C≡CH (Sebbar *et al.*, 2017*c* ▸). In the majority of these, the heterocyclic ring is quite non-planar with the dihedral angle between the plane defined by the benzene ring plus the nitro­gen and sulfur atoms and that defined by nitro­gen and sulfur and the other two carbon atoms separating them ranging from ca. 29 (**IIe**) to 36° (**IId**). The other three (**IIa**, **IIc**, **IIf**) have the benzo­thia­zine unit nearly planar with the corresponding dihedral angle of *ca* 3–4°. In the case of **IIa**, the displacement ellipsoid for the sulfur atom shows a considerable elongation perpendicular to the mean plane of the heterocyclic ring, suggesting disorder, and a greater degree of non-planarity but for the other two, there is no obvious source for the near planarity.
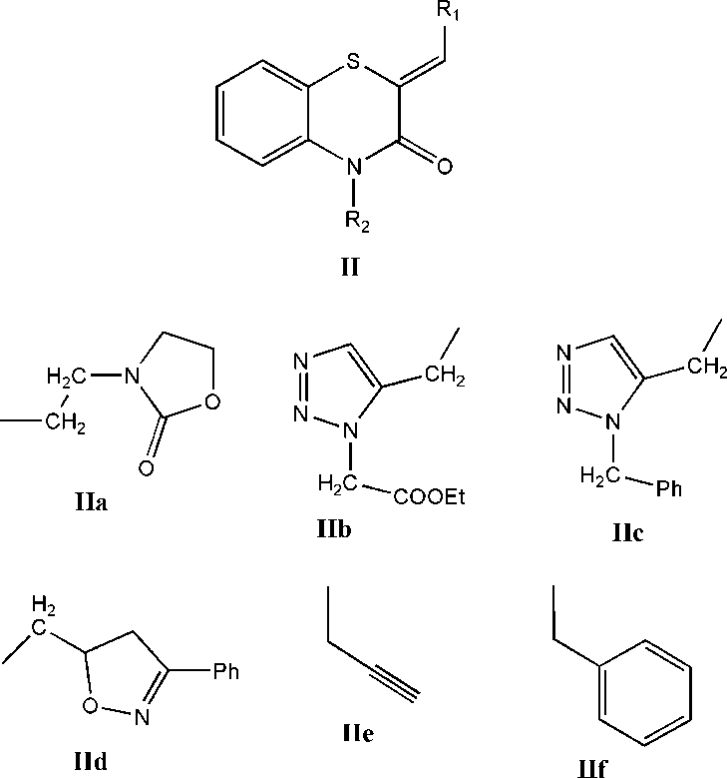


## Synthesis and crystallization   

Propargyl bromide (4 mmol) was added to a mixture of (*Z*)-2-(4-fluoro­benzyl­idene)-2*H*-1,4-benzo­thia­zin-3(4*H*)-one (1.6 mmol), potassium carbonate (4 mmol) and tetra-*n*-butyl ammonium bromide (0.15 mmol) in DMF (20 ml). Stirring was continued at room temperature for 24 h. The salts were removed by filtration and the filtrate was concentrated under reduced pressure. The residue was separated by chromatography on a column of silica gel with ethyl acetate–hexane (2/8) as eluent. The solid product obtained was recrystallized from ethanol to afford colourless crystals (yield: 89%).

## Refinement   

Crystal data, data collection and structure refinement details are summarized in Table 3 ▸. Hydrogen atoms were located in a difference-Fourier map and freely refined.

## Supplementary Material

Crystal structure: contains datablock(s) I, global. DOI: 10.1107/S2056989019002354/lh5893sup1.cif

Structure factors: contains datablock(s) I. DOI: 10.1107/S2056989019002354/lh5893Isup2.hkl

Supporting information file. DOI: 10.1107/S2056989019002354/lh5893Isup3.cdx

Supporting information file. DOI: 10.1107/S2056989019002354/lh5893Isup6.cml

CCDC reference: 1897371

Additional supporting information:  crystallographic information; 3D view; checkCIF report

## Figures and Tables

**Figure 3:**
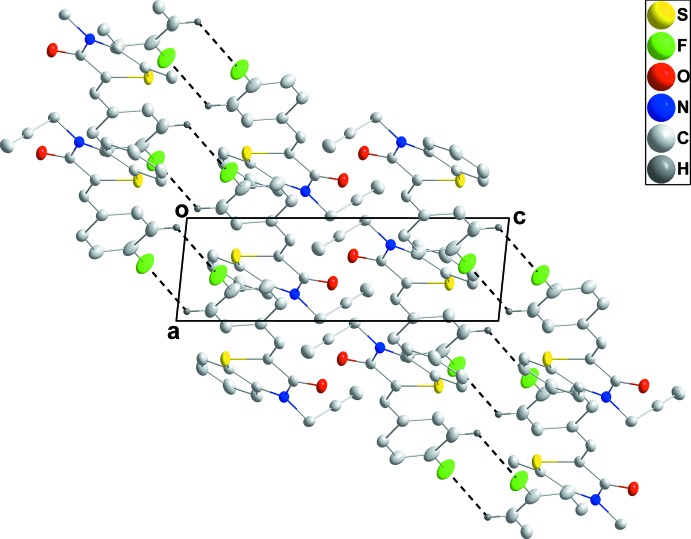
A partial packing diagram viewed along the *b*-axis direction. The inter­molecular C—H_Flurphen_⋯F_Flurphen_ (Flurphen = fluoro­phen­yl) hydrogen bonds are shown as dashed lines.

**Table 1:** Hydrogen-bond geometry (Å, °)

*D*—H⋯*A*	*D*—H	H⋯*A*	*D*⋯*A*	*D*—H⋯*A*
C15—H15⋯F1^ii^	0.98 (2)	2.60 (2)	3.306 (2)	128.5 (17)

Symmetry code: (ii) 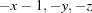.

**Table 2:** Selected interatomic distances (Å)

S1⋯N1	3.0702 (15)	C10⋯C11^vii^	3.572 (3)
S1⋯C14	3.179 (2)	C12⋯C18^vii^	3.343 (3)
S1⋯C2^i^	3.5158 (19)	C13⋯C18^vii^	3.464 (2)
S1⋯H14	2.51 (3)	C13⋯C17^vii^	3.439 (3)
S1⋯H2^i^	3.06 (2)	C14⋯C17^vii^	3.404 (3)
F1⋯F1^ii^	3.051 (2)	C14⋯C16^vii^	3.457 (3)
F1⋯C15^ii^	3.306 (3)	C15⋯C16^vii^	3.495 (3)
F1⋯H4^iii^	2.59 (3)	C4⋯H11^viii^	2.91 (3)
F1⋯H15^ii^	2.60 (2)	C5⋯H9*B*	2.63 (2)
O1⋯C10	3.167 (3)	C5⋯H11^viii^	2.80 (3)
O1⋯C18^iv^	3.388 (2)	C6⋯H9*B* ^vi^	2.85 (2)
O1⋯C18^v^	3.261 (2)	C7⋯H9*A* ^vi^	2.81 (2)
O1⋯H12	2.33 (2)	C8⋯H14	2.97 (2)
O1⋯H9*A* ^vi^	2.83 (2)	C9⋯H5	2.48 (3)
O1⋯H9*A*	2.26 (2)	C10⋯H5	2.60 (2)
O1⋯H12^v^	2.70 (2)	C10⋯H9*B* ^vi^	2.90 (2)
O1⋯H18^iv^	2.60 (2)	C11⋯H5^ix^	2.81 (3)
O1⋯H18^v^	2.71 (2)	C11⋯H9*B* ^vi^	2.99 (2)
N1⋯H9*B* ^vi^	2.85 (2)	C11⋯H17^iv^	2.82 (3)
C5⋯C10	3.216 (2)	H2⋯H2^x^	2.57 (4)
C7⋯C12^vii^	3.448 (3)	H5⋯H9*B*	2.17 (3)
C7⋯C9^vi^	3.334 (3)	H5⋯H11^viii^	2.52 (4)
C9⋯C10^vii^	3.504 (2)	H9*A*⋯H18^v^	2.50 (3)
C9⋯C11^vii^	3.469 (3)	H12⋯H18	2.32 (3)

Symmetry codes: (i) 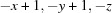; (ii) 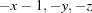; (iii) 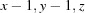; (iv) 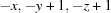; (v) 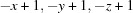; (vi) 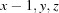; (vii) 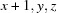; (viii) 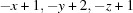; (ix) 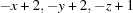; (x) 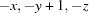.

**Table 3:** Experimental details

Crystal data	
Chemical formula	C_18_H_12_FNOS
*M*_r_	309.35
Crystal system, space group	Triclinic, *P* 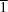
Temperature (K)	150
*a*, *b*, *c* (Å)	4.0602 (2), 13.8983 (5), 14.2620 (5)
α, β, γ (°)	117.809 (2), 93.155 (2), 94.416 (2)
*V* (Å^3^)	705.96 (5)
*Z*	2
Radiation type	Cu *K*α
μ (mm^−1^)	2.15
Crystal size (mm)	0.45 × 0.21 × 0.01

Data collection	
Diffractometer	Bruker D8 VENTURE PHOTON 100 CMOS
Absorption correction	Numerical (*SADABS*; Krause *et al.*, 2015 ▸)
*T*_min_, *T*_max_	0.69, 0.97
No. of measured, independent and observed [*I* > 2σ(*I*)] reflections	5323, 2595, 2256
*R*_int_	0.026
(sin θ/λ)_max_ (Å^−1^)	0.618

Refinement	
*R*[*F* ^2^ > 2σ(*F* ^2^)], *wR*(*F* ^2^), *S*	0.036, 0.092, 1.04
No. of reflections	2595
No. of parameters	247
H-atom treatment	All H-atom parameters refined
Δρ_max_, Δρ_min_ (e Å^−3^)	0.23, −0.31

Computer programs: *APEX3* and *SAINT* (Bruker, 2016 ▸), *SHELXT* (Sheldrick, 2015*a* ▸), *SHELXL2018* (Sheldrick, 2015*b* ▸), *DIAMOND* (Brandenburg & Putz, 2012 ▸) and *SHELXTL* (Sheldrick, 2008 ▸).

## supplementary crystallographic information

### Crystal data

**Table:**

C_18_H_12_FNOS	*Z* = 2
*M**_r_* = 309.35	*F*(000) = 320
Triclinic, *P*1	*D*_x_ = 1.455 Mg m^−^^3^
*a* = 4.0602 (2) Å	Cu *K*α radiation, λ = 1.54178 Å
*b* = 13.8983 (5) Å	Cell parameters from 4191 reflections
*c* = 14.2620 (5) Å	θ = 3.6–72.3°
α = 117.809 (2)°	µ = 2.15 mm^−^^1^
β = 93.155 (2)°	*T* = 150 K
γ = 94.416 (2)°	Plate, light yellow
*V* = 705.96 (5) Å^3^	0.45 × 0.21 × 0.01 mm

### Data collection

**Table:**

Bruker D8 VENTURE PHOTON 100 CMOS diffractometer	2595 independent reflections
Radiation source: INCOATEC IµS micro-focus source	2256 reflections with *I* > 2σ(*I*)
Mirror monochromator	*R*_int_ = 0.026
Detector resolution: 10.4167 pixels mm^-1^	θ_max_ = 72.2°, θ_min_ = 3.6°
ω scans	*h* = −4→4
Absorption correction: numerical (*SADABS*; Krause *et al.*, 2015)	*k* = −17→15
*T*_min_ = 0.69, *T*_max_ = 0.97	*l* = −15→17
5323 measured reflections	

### Refinement

**Table:**

Refinement on *F*^2^	Primary atom site location: structure-invariant direct methods
Least-squares matrix: full	Secondary atom site location: difference Fourier map
*R*[*F*^2^ > 2σ(*F*^2^)] = 0.036	Hydrogen site location: difference Fourier map
*wR*(*F*^2^) = 0.092	All H-atom parameters refined
*S* = 1.04	*w* = 1/[σ^2^(*F*_o_^2^) + (0.047*P*)^2^ + 0.2564*P*] where *P* = (*F*_o_^2^ + 2*F*_c_^2^)/3
2595 reflections	(Δ/σ)_max_ < 0.001
247 parameters	Δρ_max_ = 0.23 e Å^−^^3^
0 restraints	Δρ_min_ = −0.31 e Å^−^^3^

### Special details

**Table:**

Geometry. All esds (except the esd in the dihedral angle between two l.s. planes) are estimated using the full covariance matrix. The cell esds are taken into account individually in the estimation of esds in distances, angles and torsion angles; correlations between esds in cell parameters are only used when they are defined by crystal symmetry. An approximate (isotropic) treatment of cell esds is used for estimating esds involving l.s. planes.
Refinement. Refinement of F^2^ against ALL reflections. The weighted R-factor wR and goodness of fit S are based on F^2^, conventional R-factors R are based on F, with F set to zero for negative F^2^. The threshold expression of F^2^ > 2sigma(F^2^) is used only for calculating R-factors(gt) etc. and is not relevant to the choice of reflections for refinement. R-factors based on F^2^ are statistically about twice as large as those based on F, and R- factors based on ALL data will be even larger.

### Fractional atomic coordinates and isotropic or equivalent isotropic displacement parameters (Å^2^)

**Table:**

	*x*	*y*	*z*	*U*_iso_*/*U*_eq_	
S1	0.36900 (11)	0.48920 (3)	0.16432 (3)	0.02650 (14)	
F1	−0.4439 (4)	0.02242 (10)	0.11539 (11)	0.0500 (4)	
O1	0.6330 (3)	0.62345 (10)	0.47251 (9)	0.0274 (3)	
N1	0.7395 (3)	0.69008 (11)	0.35897 (11)	0.0196 (3)	
C1	0.4638 (4)	0.62082 (14)	0.17880 (13)	0.0216 (3)	
C2	0.3701 (5)	0.63770 (16)	0.09280 (14)	0.0280 (4)	
H2	0.244 (6)	0.5760 (19)	0.0305 (18)	0.040 (6)*	
C3	0.4589 (5)	0.73747 (16)	0.09560 (15)	0.0302 (4)	
H3	0.400 (5)	0.7482 (18)	0.0363 (18)	0.035 (6)*	
C4	0.6386 (5)	0.82155 (16)	0.18618 (16)	0.0309 (4)	
H4	0.717 (5)	0.8918 (19)	0.1894 (17)	0.037 (6)*	
C5	0.7262 (4)	0.80684 (15)	0.27349 (14)	0.0253 (4)	
H5	0.838 (6)	0.8665 (19)	0.3380 (18)	0.034 (6)*	
C6	0.6440 (4)	0.70567 (14)	0.27073 (13)	0.0200 (3)	
C7	0.5847 (4)	0.61287 (13)	0.38287 (13)	0.0201 (3)	
C8	0.3693 (4)	0.51633 (13)	0.29720 (13)	0.0200 (3)	
C9	0.9780 (4)	0.77412 (14)	0.44531 (14)	0.0226 (4)	
H9A	1.078 (5)	0.7384 (17)	0.4841 (16)	0.027 (5)*	
H9B	1.157 (5)	0.7991 (17)	0.4139 (16)	0.029 (5)*	
C10	0.8244 (4)	0.86876 (14)	0.52130 (13)	0.0232 (4)	
C11	0.7008 (5)	0.94494 (16)	0.58223 (16)	0.0323 (4)	
H11	0.598 (7)	1.005 (2)	0.627 (2)	0.058 (8)*	
C12	0.2140 (4)	0.44701 (14)	0.32673 (13)	0.0221 (4)	
H12	0.239 (5)	0.4694 (17)	0.4023 (17)	0.027 (5)*	
C13	0.0273 (4)	0.33862 (14)	0.26424 (13)	0.0224 (4)	
C14	0.0509 (5)	0.26864 (15)	0.15586 (14)	0.0273 (4)	
H14	0.190 (5)	0.2934 (18)	0.1155 (17)	0.033 (6)*	
C15	−0.1081 (5)	0.16234 (16)	0.10546 (16)	0.0317 (4)	
H15	−0.091 (6)	0.1127 (19)	0.0299 (19)	0.041 (6)*	
C16	−0.2926 (5)	0.12750 (16)	0.16410 (17)	0.0337 (4)	
C17	−0.3307 (5)	0.19315 (17)	0.26970 (16)	0.0334 (4)	
H17	−0.461 (6)	0.166 (2)	0.3075 (19)	0.046 (7)*	
C18	−0.1680 (4)	0.29876 (15)	0.31937 (15)	0.0258 (4)	
H18	−0.184 (5)	0.3440 (17)	0.3957 (18)	0.029 (5)*	

### Atomic displacement parameters (Å^2^)

**Table:**

	*U*^11^	*U*^22^	*U*^33^	*U*^12^	*U*^13^	*U*^23^
S1	0.0385 (3)	0.0213 (2)	0.0162 (2)	−0.00484 (17)	0.00200 (16)	0.00729 (18)
F1	0.0686 (9)	0.0280 (6)	0.0495 (8)	−0.0221 (6)	−0.0186 (6)	0.0217 (6)
O1	0.0346 (7)	0.0275 (7)	0.0186 (6)	−0.0014 (5)	−0.0013 (5)	0.0108 (5)
N1	0.0214 (7)	0.0177 (7)	0.0176 (7)	0.0015 (5)	0.0016 (5)	0.0067 (6)
C1	0.0221 (8)	0.0235 (9)	0.0208 (8)	0.0027 (6)	0.0047 (6)	0.0114 (7)
C2	0.0297 (10)	0.0330 (10)	0.0214 (9)	0.0033 (7)	0.0040 (7)	0.0129 (8)
C3	0.0378 (11)	0.0351 (11)	0.0255 (9)	0.0094 (8)	0.0076 (7)	0.0197 (9)
C4	0.0426 (11)	0.0257 (10)	0.0311 (10)	0.0083 (8)	0.0130 (8)	0.0173 (9)
C5	0.0291 (9)	0.0214 (9)	0.0247 (9)	0.0035 (7)	0.0070 (7)	0.0097 (8)
C6	0.0190 (8)	0.0212 (8)	0.0195 (8)	0.0044 (6)	0.0055 (6)	0.0087 (7)
C7	0.0214 (8)	0.0202 (8)	0.0182 (8)	0.0054 (6)	0.0032 (6)	0.0080 (7)
C8	0.0220 (8)	0.0178 (8)	0.0188 (8)	0.0030 (6)	0.0025 (6)	0.0073 (7)
C9	0.0198 (8)	0.0208 (8)	0.0229 (8)	−0.0001 (6)	−0.0004 (6)	0.0075 (7)
C10	0.0225 (8)	0.0215 (9)	0.0227 (8)	−0.0046 (6)	−0.0010 (6)	0.0095 (7)
C11	0.0336 (11)	0.0231 (10)	0.0321 (10)	−0.0004 (8)	0.0083 (8)	0.0064 (9)
C12	0.0259 (9)	0.0224 (9)	0.0182 (8)	0.0044 (6)	0.0044 (6)	0.0092 (7)
C13	0.0229 (8)	0.0214 (9)	0.0248 (9)	0.0011 (6)	−0.0006 (6)	0.0131 (7)
C14	0.0338 (10)	0.0230 (9)	0.0248 (9)	0.0007 (7)	0.0035 (7)	0.0115 (8)
C15	0.0413 (11)	0.0219 (9)	0.0271 (10)	−0.0005 (8)	−0.0038 (8)	0.0088 (8)
C16	0.0401 (11)	0.0216 (9)	0.0393 (11)	−0.0087 (8)	−0.0132 (8)	0.0178 (8)
C17	0.0338 (10)	0.0346 (11)	0.0381 (11)	−0.0079 (8)	−0.0064 (8)	0.0253 (10)
C18	0.0273 (9)	0.0293 (10)	0.0252 (9)	0.0007 (7)	−0.0013 (7)	0.0173 (8)

### Geometric parameters (Å, º)

**Table:**

S1—C8	1.7511 (17)	C8—C12	1.348 (2)
S1—C1	1.7515 (17)	C9—C10	1.471 (2)
F1—C16	1.364 (2)	C9—H9A	0.99 (2)
O1—C7	1.219 (2)	C9—H9B	0.99 (2)
N1—C7	1.387 (2)	C10—C11	1.183 (3)
N1—C6	1.412 (2)	C11—H11	0.93 (3)
N1—C9	1.473 (2)	C12—C13	1.461 (2)
C1—C2	1.391 (2)	C12—H12	0.97 (2)
C1—C6	1.401 (2)	C13—C18	1.400 (2)
C2—C3	1.387 (3)	C13—C14	1.404 (2)
C2—H2	0.98 (2)	C14—C15	1.388 (3)
C3—C4	1.387 (3)	C14—H14	0.98 (2)
C3—H3	0.95 (2)	C15—C16	1.373 (3)
C4—C5	1.385 (3)	C15—H15	0.98 (2)
C4—H4	0.98 (2)	C16—C17	1.375 (3)
C5—C6	1.402 (2)	C17—C18	1.386 (3)
C5—H5	0.96 (2)	C17—H17	0.95 (2)
C7—C8	1.493 (2)	C18—H18	0.98 (2)
			
S1···N1	3.0702 (15)	C10···C11^vii^	3.572 (3)
S1···C14	3.179 (2)	C12···C18^vii^	3.343 (3)
S1···C2^i^	3.5158 (19)	C13···C18^vii^	3.464 (2)
S1···H14	2.51 (3)	C13···C17^vii^	3.439 (3)
S1···H2^i^	3.06 (2)	C14···C17^vii^	3.404 (3)
F1···F1^ii^	3.051 (2)	C14···C16^vii^	3.457 (3)
F1···C15^ii^	3.306 (3)	C15···C16^vii^	3.495 (3)
F1···H4^iii^	2.59 (3)	C4···H11^viii^	2.91 (3)
F1···H15^ii^	2.60 (2)	C5···H9B	2.63 (2)
O1···C10	3.167 (3)	C5···H11^viii^	2.80 (3)
O1···C18^iv^	3.388 (2)	C6···H9B^vi^	2.85 (2)
O1···C18^v^	3.261 (2)	C7···H9A^vi^	2.81 (2)
O1···H12	2.33 (2)	C8···H14	2.97 (2)
O1···H9A^vi^	2.83 (2)	C9···H5	2.48 (3)
O1···H9A	2.26 (2)	C10···H5	2.60 (2)
O1···H12^v^	2.70 (2)	C10···H9B^vi^	2.90 (2)
O1···H18^iv^	2.60 (2)	C11···H5^ix^	2.81 (3)
O1···H18^v^	2.71 (2)	C11···H9B^vi^	2.99 (2)
N1···H9B^vi^	2.85 (2)	C11···H17^iv^	2.82 (3)
C5···C10	3.216 (2)	H2···H2^x^	2.57 (4)
C7···C12^vii^	3.448 (3)	H5···H9B	2.17 (3)
C7···C9^vi^	3.334 (3)	H5···H11^viii^	2.52 (4)
C9···C10^vii^	3.504 (2)	H9A···H18^v^	2.50 (3)
C9···C11^vii^	3.469 (3)	H12···H18	2.32 (3)
			
C8—S1—C1	101.73 (8)	C10—C9—H9A	108.6 (12)
C7—N1—C6	125.59 (14)	N1—C9—H9A	106.8 (12)
C7—N1—C9	114.59 (13)	C10—C9—H9B	109.9 (12)
C6—N1—C9	118.68 (14)	N1—C9—H9B	109.3 (12)
C2—C1—C6	120.19 (16)	H9A—C9—H9B	108.7 (17)
C2—C1—S1	117.64 (14)	C11—C10—C9	179.8 (2)
C6—C1—S1	122.07 (13)	C10—C11—H11	177.1 (17)
C3—C2—C1	120.78 (17)	C8—C12—C13	131.55 (16)
C3—C2—H2	122.1 (14)	C8—C12—H12	115.9 (12)
C1—C2—H2	117.1 (14)	C13—C12—H12	112.4 (12)
C4—C3—C2	119.25 (17)	C18—C13—C14	117.96 (16)
C4—C3—H3	120.1 (14)	C18—C13—C12	116.92 (16)
C2—C3—H3	120.7 (14)	C14—C13—C12	124.90 (16)
C5—C4—C3	120.61 (17)	C15—C14—C13	121.06 (17)
C5—C4—H4	117.9 (13)	C15—C14—H14	118.9 (13)
C3—C4—H4	121.4 (13)	C13—C14—H14	119.9 (13)
C4—C5—C6	120.64 (17)	C16—C15—C14	118.26 (18)
C4—C5—H5	120.7 (14)	C16—C15—H15	120.2 (14)
C6—C5—H5	118.6 (14)	C14—C15—H15	121.5 (14)
C1—C6—C5	118.49 (16)	F1—C16—C15	118.42 (19)
C1—C6—N1	120.91 (15)	F1—C16—C17	118.38 (18)
C5—C6—N1	120.60 (15)	C15—C16—C17	123.20 (18)
O1—C7—N1	119.59 (15)	C16—C17—C18	117.97 (18)
O1—C7—C8	121.15 (15)	C16—C17—H17	120.7 (15)
N1—C7—C8	119.23 (14)	C18—C17—H17	121.4 (15)
C12—C8—C7	116.29 (15)	C17—C18—C13	121.53 (18)
C12—C8—S1	123.30 (13)	C17—C18—H18	117.9 (12)
C7—C8—S1	119.93 (12)	C13—C18—H18	120.5 (12)
C10—C9—N1	113.47 (14)		
			
C8—S1—C1—C2	−157.17 (14)	N1—C7—C8—C12	−177.04 (15)
C8—S1—C1—C6	26.38 (15)	O1—C7—C8—S1	−167.49 (13)
C6—C1—C2—C3	1.4 (3)	N1—C7—C8—S1	10.7 (2)
S1—C1—C2—C3	−175.12 (14)	C1—S1—C8—C12	159.13 (15)
C1—C2—C3—C4	−1.1 (3)	C1—S1—C8—C7	−29.17 (15)
C2—C3—C4—C5	−0.6 (3)	C7—N1—C9—C10	−87.21 (18)
C3—C4—C5—C6	2.0 (3)	C6—N1—C9—C10	81.32 (18)
C2—C1—C6—C5	0.0 (2)	C7—C8—C12—C13	−169.96 (16)
S1—C1—C6—C5	176.34 (13)	S1—C8—C12—C13	2.0 (3)
C2—C1—C6—N1	179.64 (15)	C8—C12—C13—C18	−165.58 (18)
S1—C1—C6—N1	−4.0 (2)	C8—C12—C13—C14	19.9 (3)
C4—C5—C6—C1	−1.7 (3)	C18—C13—C14—C15	−1.5 (3)
C4—C5—C6—N1	178.67 (16)	C12—C13—C14—C15	172.96 (17)
C7—N1—C6—C1	−23.7 (2)	C13—C14—C15—C16	0.7 (3)
C9—N1—C6—C1	169.12 (14)	C14—C15—C16—F1	−178.73 (17)
C7—N1—C6—C5	155.92 (16)	C14—C15—C16—C17	0.8 (3)
C9—N1—C6—C5	−11.2 (2)	F1—C16—C17—C18	178.11 (17)
C6—N1—C7—O1	−162.11 (15)	C15—C16—C17—C18	−1.5 (3)
C9—N1—C7—O1	5.5 (2)	C16—C17—C18—C13	0.6 (3)
C6—N1—C7—C8	19.7 (2)	C14—C13—C18—C17	0.8 (3)
C9—N1—C7—C8	−172.72 (14)	C12—C13—C18—C17	−174.05 (16)
O1—C7—C8—C12	4.8 (2)		

Symmetry codes: (i) −*x*+1, −*y*+1, −*z*; (ii) −*x*−1, −*y*, −*z*; (iii) *x*−1, *y*−1, *z*; (iv) −*x*, −*y*+1, −*z*+1; (v) −*x*+1, −*y*+1, −*z*+1; (vi) *x*−1, *y*, *z*; (vii) *x*+1, *y*, *z*; (viii) −*x*+1, −*y*+2, −*z*+1; (ix) −*x*+2, −*y*+2, −*z*+1; (x) −*x*, −*y*+1, −*z*.

### Hydrogen-bond geometry (Å, º)

**Table:**

*D*—H···*A*	*D*—H	H···*A*	*D*···*A*	*D*—H···*A*
C15—H15···F1^ii^	0.98 (2)	2.60 (2)	3.306 (2)	128.5 (17)

Symmetry code: (ii) −*x*−1, −*y*, −*z*.
